# Wettability-Enhanced SiC–Graphite Synergy in Al_2_O_3_-SiC-C Castables: Carbon Resource Comparation, Sintering Response, and Latent Rheology Effects

**DOI:** 10.3390/ma18153618

**Published:** 2025-07-31

**Authors:** Benjun Cheng, Mingyang Huang, Guoqi Liu, Feng Wu, Xiaocheng Liang

**Affiliations:** 1 School of Energy Science and Engineering, Central South University, Changsha 410083, China; chbj666@csu.edu.cn (B.C.); 243912066@csu.edu.cn (M.H.); 2 School of Materials Science and Engineering, Shanghai University, Shanghai 200444, China; liuguoqi@shu.edu.cn; 3 School of Metallurgy and Materials, University of Science and Technology Liaoning, Anshan 114051, China; wufeng@ustl.edu.cn

**Keywords:** Al_2_O_3_-SiC-C castable, modified flake graphite, carbon resource, erosion resistance

## Abstract

Research on raw materials for Al_2_O_3_-SiC-C refractory castables used in blast furnace troughs is relatively well established. However, gaps remain in both laboratory and industrial trials concerning the performance of castables incorporating SiC-modified flake graphite and alternative carbon sources. This study investigated the sintering behavior, mechanical properties, and service performance of Al_2_O_3_-SiC-C castables utilizing varying contents of modified flake graphite, pitch, and carbon black as carbon sources. Samples were characterized using SEM, XRD, and EDS for phase composition and microstructural morphology analysis. Key findings revealed that the thermal expansion mismatch between the SiC coating and flake graphite in SiC-modified graphite generated a microcrack-toughening effect. This effect, combined with the synergistic reinforcement from both components, enhanced the mechanical properties. The SiC modification layer improved the wettability and oxidation resistance of the flake graphite. This modified graphite further contributed to enhanced erosion resistance through mechanisms of matrix pinning and crack deflection within the microstructure. However, the microcracks induced by thermal mismatch concurrently reduced erosion resistance, resulting in an overall limited net improvement in erosion resistance attributable to the modified graphite. Specimens containing 1 wt.% modified flake graphite exhibited the optimal overall performance. During industrial trials, this formulation unexpectedly demonstrated a water reduction mechanism requiring further investigation.

## 1. Introduction

Blast furnace troughs are critical equipment in the iron and steel industry, primarily used to channel and transport high-temperature molten iron discharged from the blast furnace. Their core function is to guide the flow of molten iron and slag, achieving slag/iron separation [1]. Analysis of the service conditions for blast furnace trough refractories indicates a harsh operating environment, necessitating the following properties: i. withstand scouring by high-temperature molten iron, requiring excellent high-temperature resistance and erosion resistance; ii. experience severe thermal fluctuations (from near-ambient temperature before iron flow to intense heat during scouring), demanding outstanding thermal shock resistance; iii. resist chemical reactions with heavy metal elements (e.g., Cu, Pb, etc.) in molten iron and acidic/alkaline compounds (e.g., CaO, SiO_2_, etc.) in slag that interact with refractory components (e.g., MnO), necessitating superior slag corrosion resistance; and iv. prevent structural degradation caused by infiltration of molten slag/iron into material pores, requiring excellent penetration resistance and low porosity [2,3,4,5].

The most commonly used refractory material for blast furnace troughs is Al_2_O_3_-SiC-C castables [6,7]. Al_2_O_3_-SiC-C refractory castables are based on alumina and carbon, meaning they are refractory castables primarily composed of alumina (as corundum) and carbon. Corundum possesses a high melting point, and carbon-containing materials exhibit high thermal conductivity; the combination of these two components endows the material with excellent high-temperature resistance and thermal shock resistance [8,9,10]. Alumina demonstrates good resistance to both basic and acidic iron slags. Optimizing the carbon-containing materials can reduce porosity; thus, this combination provides good chemical stability. Simultaneously, the addition of carbon-containing materials enhances the toughness of Al_2_O_3_-SiC-C refractories, giving them Hot Modulus of Rupture (HMOR) and erosion resistance [11,12,13]. In addition, the interaction between Al_2_O_3_-SiC-C refractories and carbon-containing materials such as graphite deserves attention [14,15,16]. However, with the development of the steel industry and the increasing demand for low-carbon steel [17], the decarburization characteristics of the ironmaking process impose higher requirements on Al_2_O_3_-SiC-C refractories. The main current technical challenge is to reduce the permeation of carbon from the refractory into the molten steel and lower its carbon content while ensuring material strength, achieved through the rational addition of modified flake graphite [18,19].

Among carbon sources for blast furnace trough refractories, graphite typically plays a dominant role. Against the backdrop of requirements for clean steel production and a low-carbon economy, graphite is a key factor in maintaining and enhancing refractory performance and is crucial for the sustainable development of the refractory industry [20]. The current graphite content in such refractories typically ranges from 5 to 30 wt.%. Structurally, the lamellar structure of graphite forms a network within the matrix, enhancing the material’s toughness and strength, particularly modulus of rupture and erosion resistance, enabling it to withstand molten iron scouring and thermal stress impact. Regarding thermal shock resistance, graphite’s low thermal expansion coefficient and high thermal conductivity help reduce stress during temperature changes and minimize crack formation. In terms of erosion resistance, graphite’s chemical inertness reduces slag penetration and may facilitate the formation of a protective layer [21,22]. Simultaneously, graphite’s excellent electrical and thermal conductivity impart outstanding thermal stability to the refractory, aiding in uniform heat dispersion under high-temperature conditions and preventing performance degradation due to localized overheating. Its superior lubricity also effectively reduces friction between internal particles, improving the material’s formability and making it easier to achieve the desired shape and structure during processing [23,24,25]. Al_2_O_3_-SiC-C refractories also incorporate other carbon-containing materials such as pitch and carbon black. Low-carbon refractories often use a phenolic resin as a binder, but the pyrolytic carbon formed after heat treatment exhibits high brittleness and poor oxidation resistance. Adding a certain amount of pitch to form a composite binder with phenolic resin results, after heat treatment, in a mixed structure of isotropic carbon and graphitic carbon. Additionally, during heating, pitch undergoes softening, melting, and solidification, tightly bonding the alumina aggregates with other components and imparting good formability and initial strength to the material [26]. Carbon black, due to its small particle size and large specific surface area, fills internal pores within the material, improving densification [27,28]. Furthermore, carbon black synergizes with other components at high temperatures to enhance the material’s oxidation resistance by forming a protective oxide layer, thereby slowing the oxidation rate [29,30].

In improving the hydrophilicity of graphite surfaces, S. Mukhopadhyay et al. [31] addressed issues in traditional alumina–carbon refractories—namely high porosity and poor slag resistance due to graphite’s hydrophobicity and the need to reduce graphite content for low-carbon requirements. They proposed preparing a calcium aluminate nanocoating on graphite surfaces via a sol–gel method to enhance interfacial hydrophilicity and oxidation resistance. To tackle the problem of carbon oxidation in graphite at high temperatures, Mula Raju et al. [32] employed a carbothermal reduction method to prepare a graphite–SiC microcomposite powder. Partially replacing flake graphite with this composite in low-carbon magnesia–carbon refractories significantly improved oxidation resistance. Abbas Ramezani et al. [33] investigated the effect of replacing calcium aluminate cement with nano-silica as a binder on the corrosion resistance of high-alumina refractory castables. They found that the nano-silica binder forms a three-dimensional network structure of Si-O-Si bonds through gelation, resulting in a uniform pore distribution after drying. This reduces molten metal penetration and eliminates the hydration–dehydration process, thereby lowering cracking risks. Z. Chen et al. [34] studied silicon-containing alumina–carbon refractories using graphite flakes and carbon black as carbon sources. They discovered that different carbon sources affect the morphology of in situ formed SiC: carbon black generates spherical SiC particles, while graphite flakes and pyrolytic carbon from the phenolic resin produce SiC whiskers.

Based on the above review, most studies focus on the water wettability of graphite surfaces and enhancing graphite’s oxidation resistance, primarily investigating how surface modification techniques affect material microstructure and mechanical properties. However, there remains a lack of in-depth exploration regarding the influence of graphite types (e.g., flake graphite), other carbon sources in Al_2_O_3_-SiC-C refractories on overall performance, and the interaction between SiC and flake graphite [35,36]. Therefore, to reduce the carbon content in Al_2_O_3_-SiC-C refractories while ensuring their strength performance under the premise of clean steel production, the key lies in rationally adjusting the proportions of flake graphite, pitch, and carbon black. For instance, during carbon reduction, decreasing the amounts of pitch and carbon black is a direct approach. However, simply reducing their addition introduces several issues: reducing pitch content weakens the material’s binding performance, leading to difficulties in forming and decreased strength; reducing carbon black content may compromise densification and lower oxidation resistance. Reducing flake graphite will inevitably result in poor wettability with water in the Al_2_O_3_-SiC-C refractory, leading to high apparent porosity and a subsequent decline in the overall strength of the refractory. Consequently, optimizing the ratio of these three components is essential to reduce carbon content while maintaining material performance.

The work explored varying proportions of flake graphite, pitch, and carbon black within Al_2_O_3_-SiC-C refractories, supplementing the understanding of how different contents of carbonaceous materials influence the properties of Al_2_O_3_-SiC-C refractories in the field.

## 2. Materials and Methods

### 2.1. Materials

The raw materials used in the experiment included brown fused alumina (Al_2_O_3_ > 96 wt.%), black silicon carbide (β-SiC > 96.6 wt.%), silica fume (average particle size: 0.15 μm, SiO_2_ > 95 wt.%), α-Al_2_O_3_ micro powder (Al_2_O_3_ > 99 wt.%, −200 mesh), pitch, carbon black (CB), modified flake graphite (MFG, C > 98 wt.%), metallic silicon powder (Si > 99 wt.%), polypropylene anti-spalling fiber (ASF), and calcium aluminate cement. The formulation compositions of the specimens are detailed in Table 1. All raw materials were sourced from Henan Hexin Refractories Co., Ltd., Luoyang, China.

### 2.2. Methods

First, raw materials were uniformly mixed according to Table 1 proportions and vibration-molded in molds. Following Chinese National Standards GB/T 3001-2007 [37] and GB/T 8931-2007 [38], specimens were prepared as follows: 40 × 40 × 160 mm for modulus of rupture (or compressive) strength testing, ф80 × 100 mm cylinders for slag erosion resistance, and 25 × 25 × 125 mm for thermal shock stability testing. A ф50 × 60 mm hole was drilled at the center of each cylindrical specimen. For thermal shock stability testing per GB/T 30873-2014 [39], the water quenching method was employed, i.e., specimens heated to 1100 °C were rapidly immersed in 20 °C water, followed by drying at 110 °C for 24 h. Modulus of rupture was then measured, and residual strength after thermal shock (R_r_) was calculated using Equation (1) to quantify thermal shock stability. For slag erosion resistance testing, 100 g of blast furnace slag (<1 mm particle size; composition: SiO_2_ 37, CaO 33, Al_2_O_3_ 13, MgO 11 wt.%) was placed into the hole of each cylindrical specimen. Specimens were heat-treated at 1400 °C for 3 h, furnace-cooled to room temperature, sectioned diametrically, and examined for erosion morphology. The erosion index (θ) was measured as a quantitative metric for erosion resistance, calculated using Equation (2).

(1) $$R_{r}=\frac{\left|R_{1}-\;R_{0}\right|}{R_{0}}\times 100\%$$

(2) $$\theta =\frac{\left|L_{1}-L_{0}\right|}{L_{0}}\times 100\%$$

where R represents the modulus rupture, L represents the depth of sample crucible, and the subscripts 0 and 1 indicate the sample before sintering and the sintered sample.

Phase analysis was performed using an X-Ray Diffractometer (XRD, D8 ADVANCE, Bruker, Berlin, Germany). XRD patterns were acquired at ambient temperature using Cu-Kα radiation, with scanning conducted over a 2θ range of 5° to 90° at a rate of 18°/min. The microstructures of heat-treated samples (polished) were examined using Field Emission Scanning Electron Microscopes (FE-SEM, MIRA4, TESCAN, Brno, Czech Republic and JSM-IT800, JEOL, Tokyo, Japan), both equipped with Energy-Dispersive Spectroscopy (EDS) systems; all polished samples were sputter-coated with gold prior to FE-SEM observation.

## 3. Results

### 3.1. Characteristics of Raw Materials

**Figure 1:**
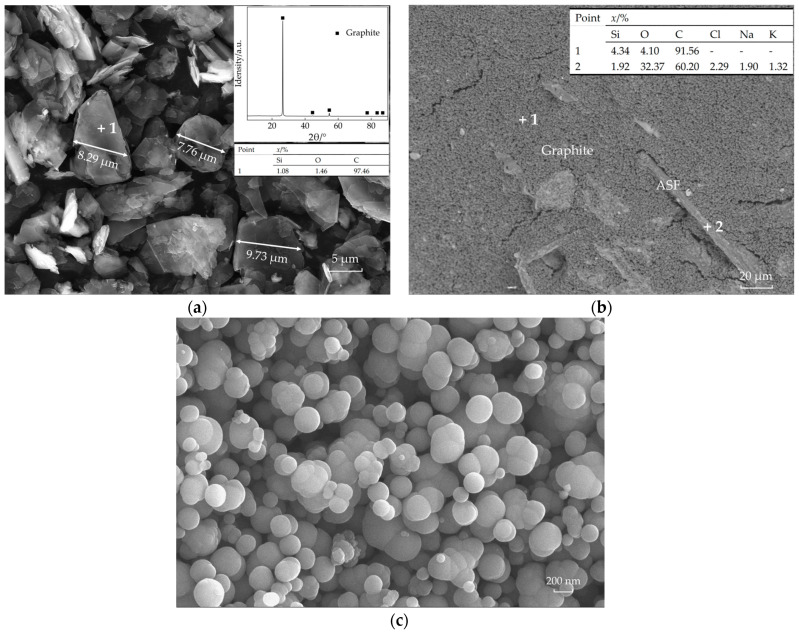
Microstructure and phase analysis of raw materials. (**a**) MFG; (**b**) MFG and ASF in the sintered sample; (**c**) carbon black.

Among the three carbon sources used, both globular pitch and CB exhibit relatively uniform spherical morphologies, while flake graphite displays a thin flake-like morphology (Figure 1a), and the CB exhibits a spherical particle morphology (Figure 1c). The pitch also presents a similar morphology like the CB. The loose structure and high viscosity of pitch significantly enhance mechanical properties but result in poor oxidation resistance. The high reactivity of CB promotes pronounced internal ceramization within the material, markedly improving densification, with thermal shock stability also showing an upward trend. The layered structure formed by stacked flake graphite in refractories effectively reduces fracture energy, thereby strengthening mechanical performance. Although no phases other than the main graphite crystalline phase appeared in the XRD patterns, it is inferred that the SiC content used for surface modification of flake graphite is extremely low, below the detection limit of 0.5 wt.%. However, the EDS results indicate trace amounts of Si, confirming that the flake graphite underwent SiC modification treatment.

Among other additives, the metallic silicon powder was incorporated to further enhance the high-temperature oxidation resistance of specimens, ensuring carbon retention post-firing to maintain functionality. ASF was added to improve explosion resistance, preventing specimen spalling during firing due to trapped steam. Trace amounts of ASF remained incompletely oxidized post-firing, with its morphology as illustrated in Figure 1b, which is from specimen M1.0.

### 3.2. Mechanical Property

**Figure 2:**
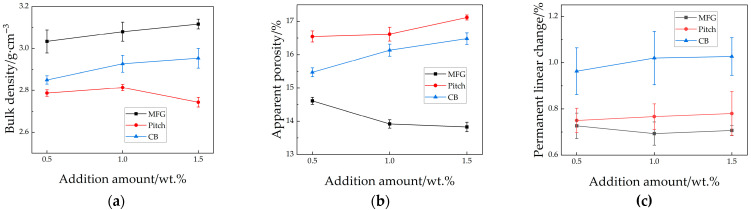
Sinter ability of samples (**a**) bulk density; (**b**) apparent porosity; (**c**) permanent linear change.

The following properties were tested for all specimens: bulk density, apparent porosity, linear change after firing, CMOR, compressive strength, HMOR, and residual strength after thermal shock. Bulk density and apparent porosity are shown in Figure 2. The sintering performance of the three specimen types ranked from the best to worst as follows: MFG > CB > pitch. With the increasing additive content, bulk density increased, and apparent porosity decreased for all specimens—except pitch-containing specimens, which exhibited a significant drop in bulk density at P1.5. Linear change after firing indicated similar dimensional stability between specimens with MFG and pitch, while those with CB showed markedly higher linear change. As additive content increased, linear change after firing rose for all specimens, though specimens with MFG exhibited minimal variation. Overall, since M1.5 showed limited improvement over M1, the 1 wt.% addition of MFG was determined as the optimal formulation considering cost-effectiveness.

**Figure 3:**
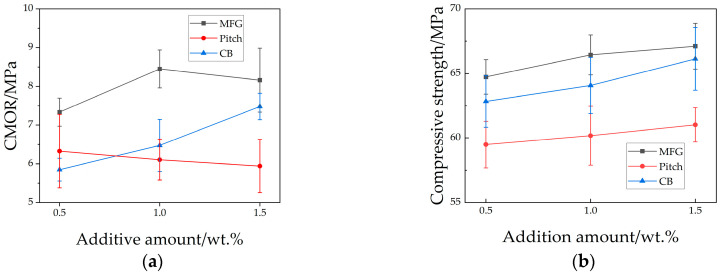
Ambient-temperature mechanical property of samples (**a**) CMOR; (**b**) compressive strength.

Results of ambient-temperature mechanical property tests are shown in Figure 3. In cross-comparisons, specimens with MFG exhibited optimal performance in both flexural and compressive strengths. While mechanical properties generally improved with increasing additive content, pitch-containing specimens demonstrated an opposite trend. Additionally, specimens with M1.5 showed slight degradation compared to lower addition levels. Overall, specimens with M1.0 achieved peak values in both strength metrics.

**Figure 4:**
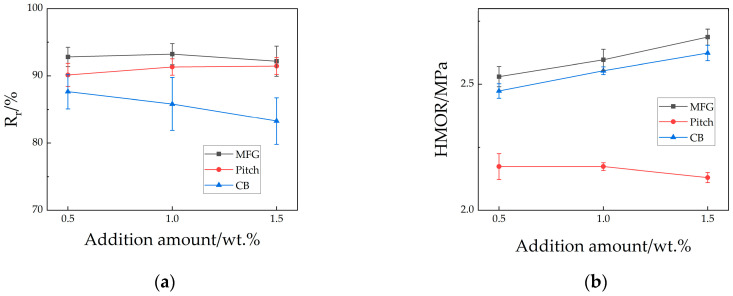
High-temperature mechanical property of samples: (**a**) residual strength after thermal shock; (**b**) HMOR.

The high-temperature mechanical property test results are shown in Figure 4. In the Hot Modulus of Rupture (HMOR), samples containing MFC and CB both performed better, showing an increasing trend with a higher additive content, while samples containing pitch showed a decreasing trend, with strength approximately 15% lower than other samples. In the thermal shock test, a reversal occurred between samples containing pitch and those containing carbon black; the latter showed a decreasing trend with increasing additive content, and its values differed significantly from those containing MFG or pitch. Samples containing flake graphite maintained residual strength above 92%, indicating superior thermal shock resistance. In summary, Al_2_O_3_-SiC-C ceramics incorporating these three carbon sources showed varying degrees of improvement in mechanical properties, with the sample M1.0 exhibiting the most superior performance in terms of sintering behavior, room-temperature properties, and high-temperature properties.

Compared with other studies [40], this study demonstrates superior performance in both comprehensive mechanical properties (with the exception of slightly lower CMOR) and thermal shock resistance.

### 3.3. Erosion Resistance and Microstructure

**Figure 5:**
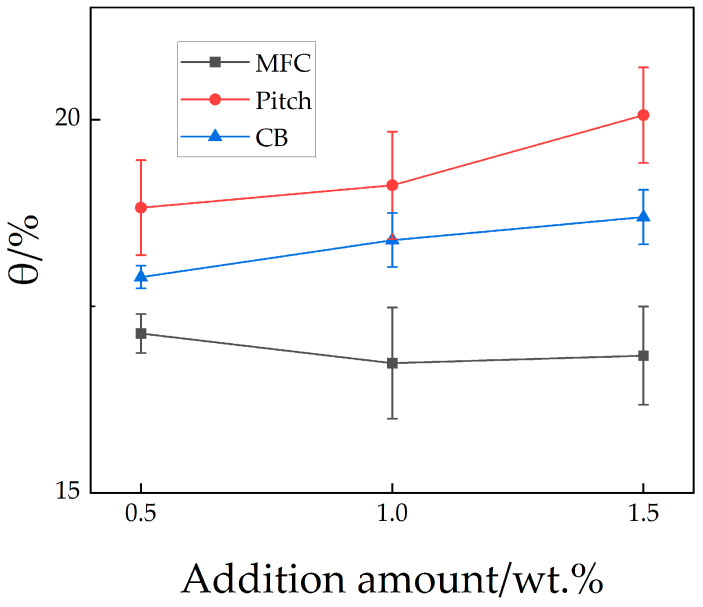
Erosion index (θ) of samples.

**Figure 6:**
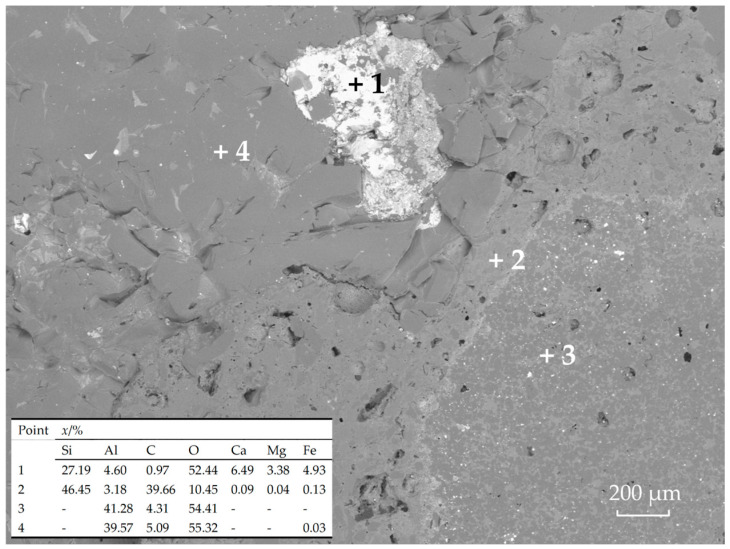
Microstructure and EDS results of erosion layer.

Slag erosion resistance test results are shown in Figure 5 and are represented by erosion index θ. The erosion resistance ranking from the best to worst was MFG > CB > pitch, with MFG specimens showing marginal superiority. Specimen M1.0 (optimal erosion resistance) was selected for microstructural observation. Figure 6 shows the microstructure at the slag penetration front. Combined with raw materials and EDS analysis, laminar dark gray areas (Point 4) are corundum aggregates; uniformly distributed dark gray spots (Point 3) represent the α-Al_2_O_3_-dominated matrix; light gray regions between them (Point 2) primarily consist of SiC and graphite; bright white zones indicate slag (Point 1). According to the EDS results, point 1 mainly contains calcium magnesium silicate and free oxides and their solid solution. Morphology reveals slag penetration along interfaces between corundum aggregates, enveloping particles and degrading the refractory structure. The dense α-Al_2_O_3_ and SiC/C matrix structurally resisted slag infiltration, with no slag observed in these regions. Notably, a distinct bright interface exists between α-Al_2_O_3_-rich and SiC/C-rich zones, inferred to be a weakly bonded interface formed by Al_2_O_3_-SiC solid solutions or mullite precursors created through SiC interdiffusion. This interface exhibited higher densification and superior high-temperature performance compared to adjacent regions [41,42], contributing to enhanced erosion resistance.

**Figure 7:**
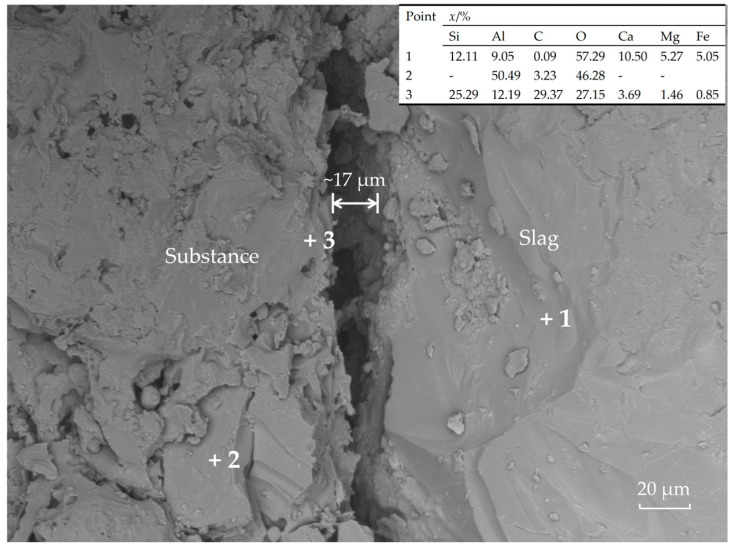
Microstructure and EDS results of interface between slag and sample.

Figure 7 shows the interfacial microstructure between localized slag and the specimen. A gap of approximately 17 μm is observed between the slag and specimen substance without adhesion, indicating poor wettability and low adhesion force between the specimen and slag. Combined with erosion patterns in Figure 6, this suggests minimal slag penetration into the specimen, enhancing erosion resistance. Only isolated slag-contact areas exist where carbon oxidation caused structural loosening. In this work, SiC encapsulation in MFG preserved graphite integrity under oxidizing conditions, further improving erosion resistance.

**Figure 8:**
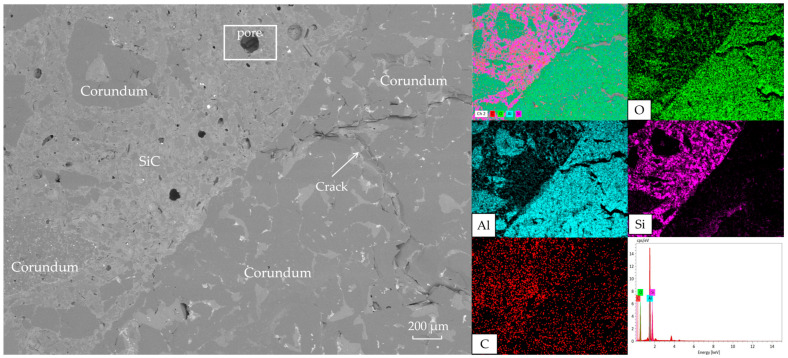
Microstructure and EDS results of the brick layer.

Figure 8 illustrates the bonding mode between aggregates and matrix. The right side shows large dark gray corundum aggregates, while light gray areas represent SiC concentrated within the matrix. Minor iron oxide and carbon impurities exist inside corundum particles. The specimen exhibits solid-phase sintering bonding. Notably, SiC on MFG surfaces provided oxidation protection during sintering. Additionally, significant macrocracks in corundum aggregates (attributed to sintering stress) terminated at aggregate–matrix interfaces, indicating stress dissipation by the matrix that prevented crack propagation.

**Figure 9:**
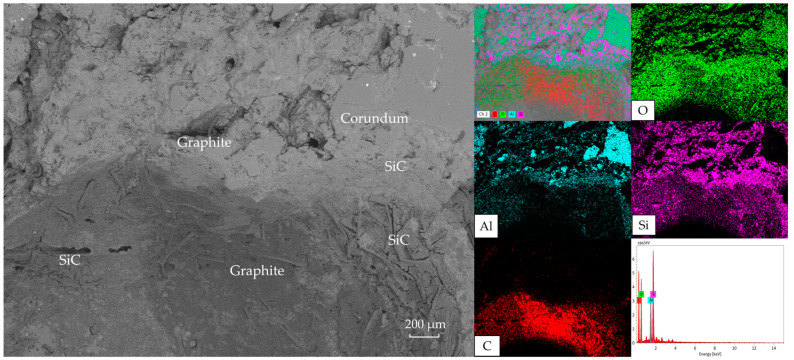
Microstructure and EDS results of graphite-rich region.

Figure 9 schematizes graphite morphology in the original brick layer. MFG maintained its lamellar structure, with minor surface voids inferred to result from Coefficient of Thermal Expansion (CTE) mismatch among SiC, graphite, and corundum [43,44,45]. The largely intact graphite morphology confirms MFG’s persistence through high-temperature sintering, enabling continuous functionality during refractory service.

## 4. Discussion

Based on the preceding results, the Al_2_O_3_-SiC-C specimens incorporating MFG demonstrated optimal performance in sintering behavior, mechanical properties, and erosion resistance (though the latter was less pronounced) [46]. Analysis of the microstructure of MFG-containing specimens reveals the following mechanisms: Firstly, the superior mechanical properties correlate with sintering performance. Despite being influenced by post-firing linear change, both CB and MFG specimens exhibited enhanced ambient-temperature flexural and compressive strength due to their high density and low porosity—high density inherently strengthens the material, while low porosity avoids providing crack propagation paths and stress concentration zones—a conclusion validated by Figure 8. This is attributed to SiO_2_ phases generated during SiC oxidation promoting liquid-phase formation, filling gaps formed during sintering. The non-wetting nature of SiC with other phases also facilitates the uniform spreading of the liquid phase. Furthermore, MFG’s high aspect ratio, synergized with the skeleton effect of surface-modified SiC, effectively absorbs or deflects crack propagation. While flake graphite oxidizes and fails at high temperatures, SiC modification improved its oxidation resistance. Figure 9 clearly shows MFG remained largely unoxidized. CB and pitch specimens exhibited inferior high-temperature mechanical properties due to CB’s high reactivity and pitch’s propensity for cracking.

Secondly, the superior thermal shock resistance of MFG specimens stems from the microcrack toughening effect induced by CTE mismatch between surface SiC and the carbon skeleton. Pitch specimens showed potential toughening from volume shrinkage due to high-temperature carbon loss, but macrocracks detached from aggregates provided propagation pathways. CB specimens, with high bulk density and densification, exhibited poor thermal shock stability as the matrix could not absorb or deflect macrocrack propagation.

Finally, slag erosion resistance is multifaceted. Micrographs indicate MFG’s superior performance primarily results from poor slag–matrix wettability, matrix-induced crack pinning and deflection, and SiC’s oxidation resistance. Figure 6 and Figure 7 collectively show primarily penetrated slag rather than the eroded specimen, implying a reduced contact area with the matrix due to poor wettability directly weakening slag penetration. Additionally, crack deflection and pinning by the matrix at aggregate interfaces (Figure 8) maintained densification, reducing slag penetration channels. The preservation of carbon (Figure 9) indicates MFG remained functional post high-temperature treatment under SiC protection—fundamental for carbon-containing refractories. Synergy of these three mechanisms gave MFG specimens a marginal edge. This marginal superiority (“slightly”) arises because CTE-mismatch microcracks between materials potentially provide penetration paths, evidenced by pores formed by MFG aggregation in Figure 9. Conversely, the isotropic, continuous nature of pitch and CB provided better physical barriers against slag penetration than anisotropic MFG. Their high reactivity also reduced slag oxidizability, positively contributing to erosion resistance. Consequently, erosion resistance differences among the three were insignificant—a direction warranting future study.

The Al_2_O_3_-SiC-C refractory with 1 wt.% MFG is undergoing continuous industrial trials at a steel plant’s tap hole. Current service life exceeds conventional materials (259 heat vs. 228 heat). Crucially, the new castable requires significantly less mixing water (3.8 wt.% vs. 5.0 wt.%), demonstrating MFG’s pronounced water-reduction effect. Elucidating MFG’s water reduction mechanism thus becomes a key future research objective, holding significant economic and environmental importance for the steel industry.

## 5. Conclusions

This study comparatively investigated the performance and engineering application of Al_2_O_3_-SiC-C refractory castables using SiC-modified flake graphite (MFG) versus other carbon sources. Sintering behavior, mechanical properties, and service performance (including thermal shock resistance and slag erosion resistance) were tested, and specimens were characterized using XRD, SEM, and EDS. Key findings are as follows:

(1) In sintering and mechanical performance testing, specimens incorporating MFG exhibited optimal performance; the liquid-phase-promoting effect and non-wettability of the SiC coating on MFG facilitated uniform liquid-phase spreading, improved sintering behavior, and reduced stress concentration under load.
(2) The slag erosion resistance of MFG-containing specimens was influenced by multiple factors: poor slag–matrix wettability, matrix-induced crack pinning and deflection, SiC’s oxidation resistance, and microcracks.
(3) In summary, the Al_2_O_3_-SiC-C castable with 1 wt.% MFG delivered optimal overall performance. This formulation underwent service testing in actual industrial conditions. Beyond the expected extended service life, a pronounced water reduction effect was observed, which will be the focus of subsequent research.

## Figures and Tables

**Table 1:** Formulas of the samples (wt.%).

Materials	P0.5	P1.0	P1.5	C0.5	C1.0	C1.5	M0.5	M1.0	M1.5
Brown fused alumina	66	66	66	66	66	66	66	66	66
Black silicon carbide	20	20	20	20	20	20	20	20	20
Silica fume	4	4	4	4	4	4	4	4	4
α-Al_2_O_3_ micro powder	3	3	3	3	3	3	3	3	3
Metallic silicon powder	1.5	1.5	1.5	1.5	1.5	1.5	1.5	1.5	1.5
ASF	1.5	1.5	1.5	1.5	1.5	1.5	1.5	1.5	1.5
Calcium aluminate cement	4	4	4	4	4	4	4	4	4
Bonds (deionized water)	+4	+4	+4	+4	+4	+4	+4	+4	+4
Pitch	+0.5	+1	+1.5	-	-	-	-	-	-
CB	-	-	-	+0.5	+1	+1.5	-	-	-
MFG	-	-	-	-	-	-	+0.5	+1	+1.5

## Data Availability

The original contributions presented in the study are included in the article. Further inquiries can be directed to the corresponding author.
